# Evaluating the Efficacy of Repetitive Transcranial Magnetic Stimulation Combined With Auditory Integration Training for Children With Autism Spectrum Disorder: Protocol for a Randomized Sham-Controlled Trial

**DOI:** 10.2196/80243

**Published:** 2026-02-11

**Authors:** Qinghong Hao, Jinying Wang, Jindi Yang, Wei Li, Sufen Hu, Zhihai Lv

**Affiliations:** 1 Department of Child Rehabilitation, Longgang District Maternity & Child Healthcare Hospital of Shenzhen City (Longgang Maternity and Child Institute of Shantou University Medical College), No. 6, Ailong Road, Longgang District, Shenzhen, 518172, China, 86 0755 28933003

**Keywords:** autism spectrum disorder, repetitive transcranial magnetic stimulation, auditory integration training, sham stimulation, randomized controlled trial

## Abstract

**Background:**

Autism spectrum disorder (ASD) represents a significant public health challenge characterized by persistent social communication deficits and restricted, repetitive patterns of behavior. Current interventions show limited efficacy, particularly for core symptoms. Repetitive transcranial magnetic stimulation (rTMS) and auditory integration training (AIT) have independently demonstrated promise in addressing neurophysiological abnormalities associated with ASD.

**Objective:**

This study aims to evaluate the clinical efficacy of a combined rTMS and AIT intervention compared to rTMS alone and sham stimulation in children with ASD.

**Methods:**

This is a randomized, sham-controlled trial that will recruit 80 children aged 2 to 6 years with a confirmed ASD diagnosis. The randomization of the first 8 participants in this study used a 1:1:1 ratio. To more effectively test the core hypothesis (ie, the efficacy of the combined intervention), greater statistical power will be concentrated on the intervention group (rTMS+AIT), and the randomization ratio was ultimately adjusted to 2:1:1—specifically, (1) rTMS combined with AIT (n=40), (2) rTMS alone (n=20), or (3) sham rTMS (n=20). Primary outcome measures include the Autism Behavior Checklist and Childhood Autism Rating Scale. Secondary outcomes are the Strengths and Difficulties Questionnaire and Repetitive Behavior Scale–Revised. Assessments will be conducted at baseline, an interim time point, and immediately after the intervention. Data will be analyzed using SPSS (version 25.0; IBM Corp).

**Results:**

This study has received funding, with data collection commencing in April 2024. Due to the small initial sample size of 8 participants (5 male and 3 female), no formal statistical comparisons of baseline characteristics between groups have been conducted at this time. It is anticipated that the rTMS combined with AIT intervention will exhibit superior efficacy compared to rTMS only.

**Conclusions:**

This will be the first sham-controlled trial to systematically investigate the potential synergistic effects of a combined rTMS and AIT intervention in children with ASD. The results will provide valuable insights into the neurotherapeutic potential of this combined approach and contribute to the development of evidence-based interventions for ASD.

## Introduction

According to the *Diagnostic and Statistical Manual of Mental Disorders, Fifth Edition*, autism spectrum disorder (ASD) is a complex neurodevelopmental condition characterized by impaired social interaction and communication as well as restricted and repetitive patterns of behavior [1,2]. Individuals with ASD may also exhibit abnormal responses to sensory stimuli, unusual emotional behaviors, difficulties with speech and language comprehension, and challenges with self-care and social adjustment [3]. The prevalence of ASD is a significant public health concern. According to the World Health Organization, approximately 1 in 100 children worldwide is diagnosed with ASD [4]. Data released by the US Centers for Disease Control and Prevention in 2023 showed a sharp increase in the prevalence of autism among children in the United States over the past 20 years [5,6]. Epidemiological survey data from a large sample population in China also indicate a prevalence rate of 0.7% for ASD [7]. Given the growing prevalence of ASD, early assessment, diagnosis, and intervention are crucial. However, the pathogenesis of ASD remains unclear, and there is a lack of specific drug treatments. It is necessary to actively explore effective clinical intervention methods to address this pressing public health challenge.

In recent years, noninvasive brain stimulation, particularly repetitive transcranial magnetic stimulation (rTMS), has been widely applied in the treatment of ASD and has gained recognition among researchers [8,9]. Transcranial magnetic stimulation (TMS) is believed to induce lasting neuroplastic changes in the brain, thereby potentially normalizing social and cognitive functioning in individuals with ASD by stabilizing abnormal neuroplasticity [10]. A meta-analysis by Smith et al [11] of 12 studies demonstrated that TMS improved social withdrawal, repetitive behaviors, and executive function in adolescents with ASD who had an IQ greater than 65, highlighting the significant therapeutic potential of TMS for ASD. Current TMS interventions for ASD primarily target the dorsolateral prefrontal cortex (DLPFC), medial prefrontal cortex, and temporo-parietal junction [12,13]. The DLPFC is involved in multiple higher-order cognitive functions, including emotional regulation, decision-making, attentional control, and adaptive adjustment. In ASD, the core symptoms of repetitive, stereotyped behaviors and social impairments are closely associated with DLPFC dysfunction. Studies using low-frequency rTMS targeting the bilateral DLPFC have demonstrated significant reductions in Repetitive Behavior Scale–Revised (RBS-R) scores following intervention [14,15]. Another study using low-frequency rTMS on the bilateral DLPFC in individuals with ASD showed significant improvements in social behavior after 18 treatment sessions. Similarly, Masuda et al [16] and Casanova et al [17] have indicated that low-frequency rTMS may exert therapeutic effects on social interaction and repetitive behaviors in ASD. In summary, DLPFC dysfunction represents a key neural mechanism underlying the core symptoms of ASD, with particularly pronounced effects on social skills and behavioral regulation. Given its favorable safety profile and emerging evidence of efficacy, rTMS offers a promising intervention for addressing the challenges faced by individuals with ASD.

Studies have shown that most children with ASD experience sensory integration dysfunction [18]. Moreover, the core symptoms of ASD are closely related to abnormal sensory processing, with auditory processing abnormalities being a primary concern [19]. Auditory integration training (AIT), a novel audio therapy initially proposed by French physician Guy Bérard [20], filters out hypersensitive frequencies to reduce auditory cortex sensitivity to sound stimuli, thereby significantly improving auditory attention in children with ASD [21]. Studies indicate that AIT effectively improves language, social interaction, communication, and behavioral skills in children with ASD who also have auditory attention deficits. International studies have explored the physiological effects of AIT on plasma glial cell–derived neurotrophic factor levels. Results demonstrate that AIT significantly elevates plasma glial cell–derived neurotrophic factor levels, thereby improving abnormal sensory processing in affected children [22]. Multiple studies support the efficacy of this method, indicating that when children with ASD can more accurately perceive and process auditory information, their comprehension and expression abilities improve, leading to enhanced functioning in social skills, communication, and related domains [18,23]. However, AIT is considered limited in its capacity to fundamentally activate and repair the nervous system, which may hinder sustained functional recovery [24]. Given this limitation, current research predominantly combines AIT with traditional behavioral training to evaluate treatment outcomes [25,26]. Furthermore, previous studies have often lacked appropriate control groups, necessitating additional research to validate AIT’s efficacy. Nevertheless, AIT can be easily implemented in laboratory or clinic settings, facilitating researchers’ collection of physiological data for objective assessment of treatment outcomes.

Although preliminary findings from these individual interventions are encouraging, the evidence base for their combined application remains limited and is subject to methodological constraints. To our knowledge, the few existing studies on combined rTMS and AIT lack adequate control conditions and are susceptible to placebo effects and observer bias [27,28]. A critical and pervasive limitation in recent studies of noninvasive brain stimulation interventions for ASD is the absence of high-quality sham-controlled trials [29,30]. This significant methodological gap prevents definitive conclusions about the true synergistic efficacy of these combined interventions. Consequently, there is an urgent need for rigorous, well-controlled research evaluating the synergistic effects of these 2 treatment modalities to provide stronger evidence-based support for ASD interventions.

The primary objective of this study is to investigate whether the combination of rTMS and AIT produces superior effects on brain activity and behavior outcomes in children with ASD compared to sham-controlled conditions. We hypothesize that the combined intervention will yield synergistic benefits, and the findings from this study are expected to provide a scientific foundation for optimizing clinical interventions for ASD.

## Methods

### Design and Setting

**Figure 1.**
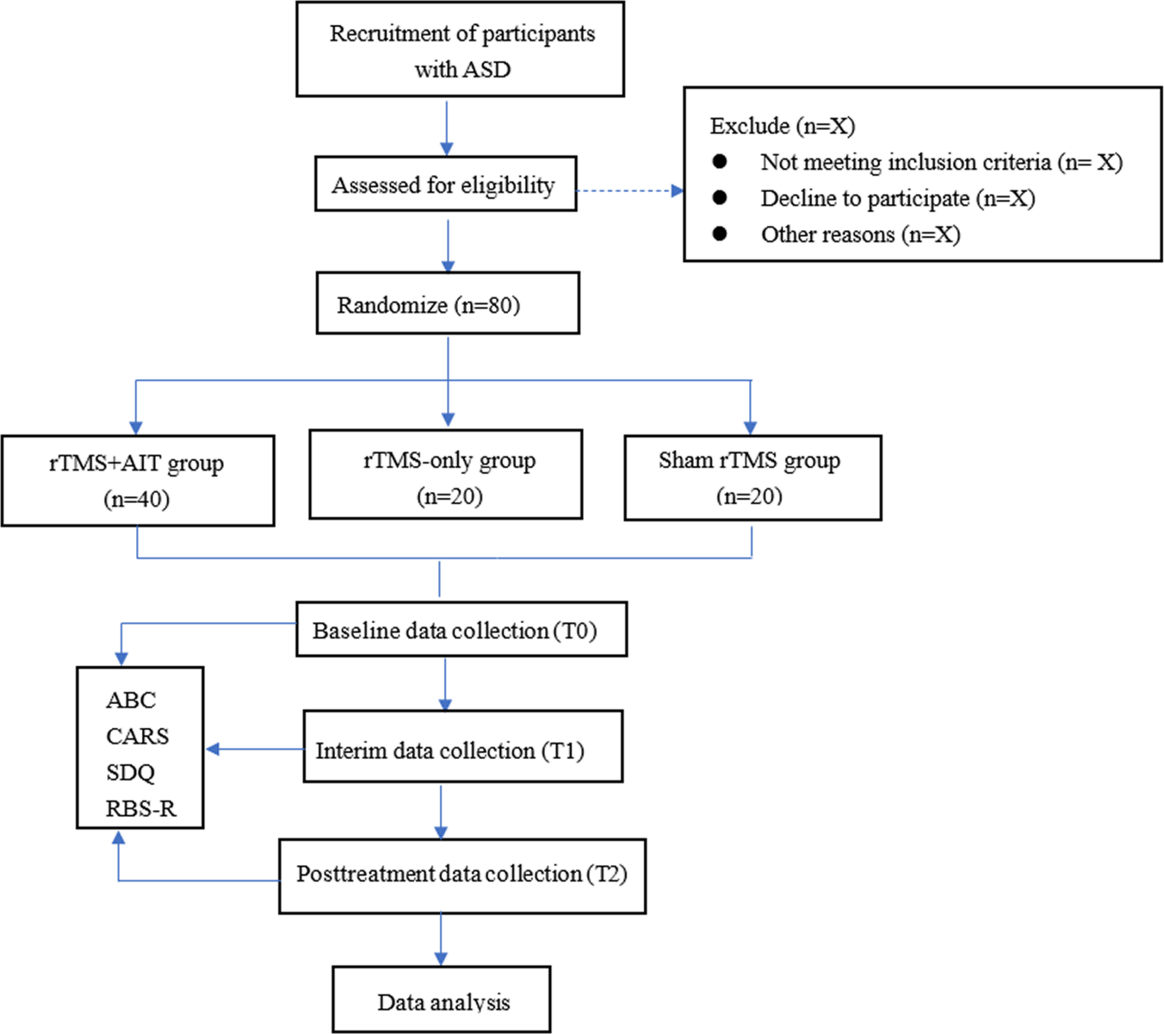
Flowchart of the trial. ABC: Autism Behavior Checklist; AIT: auditory integration training; ASD: autism spectrum disorder; CARS: Childhood Autism Rating Scale; RBS-R: Repetitive Behavior Scale–Revised; rTMS: repetitive transcranial magnetic stimulation; SDQ: Strengths and Difficulties Questionnaire.

This is a randomized, parallel-group, sham-controlled trial conducted at the Shenzhen Longgang District Maternal and Child Health Hospital. The flowchart of the experimental design is shown in Figure 1, and the schedule of participant assessments is shown in Table 1. Specifically, participants are randomly assigned in a 2:1:1 ratio to 1 of 3 intervention groups: rTMS combined with AIT, rTMS alone, or sham stimulation. Sham stimulation is used via a “sham coil” with an identical appearance to that of the active TMS coil. Participants, outcome assessors, treating therapists, data analysts, and other study personnel are kept blind to group allocation throughout the trial.

**Table 1.** Schedule of the participant assessments.

	Study period									
	Baseline (week –1)	Allocation (week 0; T0)	Treatment phase—left DLPFC^a^			Interim time point (T1)	Treatment phase—right DLPFC			Posttreatment time point (week 13; T2)
			Weeks 1-2	Weeks 3-4	Weeks 5-6		Weeks 7-8	Weeks 9-10	Weeks 11-12	
Enrollment										
Eligibility screen	✓									
Informed consent	✓									
Randomization		✓								
Interventions										
rTMS^b^+AIT^c^ group			✓	✓	✓		✓	✓	✓	
rTMS group			✓	✓	✓		✓	✓	✓	
Sham rTMS group			✓	✓	✓		✓	✓	✓	
Assessment										
ABC^d^		✓				✓				✓
CARS^e^		✓				✓				✓
SDQ^f^		✓				✓				✓
RBS-R^g^		✓				✓				✓

a DLPFC: dorsolateral prefrontal cortex.

b rTMS: repetitive transcranial magnetic stimulation.

c AIT: auditory integration training.

d ABC: Autism Behavior Checklist.

e CARS: Childhood Autism Rating Scale.

f SDQ: Strengths and Difficulties Questionnaire.

g RBS-R: Repetitive Behavior Scale–Revised.

### Participants

We are recruiting participants from inpatient and outpatient populations of the Children’s Rehabilitation Department of Shenzhen Longgang District Maternal and Child Health Hospital. Recruitment is being conducted through structured screening procedures. During recruitment, gender is not a restriction. If the proportion is unbalanced, the sample size will need to be increased.

#### Eligibility Criteria

##### Inclusion Criteria

Participants who meet the following criteria are included:

- Fulfillment of the diagnostic criteria for ASD according to the *Diagnostic and Statistical Manual of Mental Disorders, Fifth Edition* [31]
- Age of 2 to 6 years (this age range represents the period of greatest neural plasticity in the developing brain) [32]
- No significant abnormalities on routine electroencephalography (EEG) examination
- Normal olfactory, visual, and auditory function, with no other physical or psychiatric comorbidities
- Legal guardian being informed of the study procedures and providing written informed consent

##### Exclusion Criteria

Participants who meet any of the following criteria are excluded:

- Significant abnormalities on cranial magnetic resonance imaging or severe cardiopulmonary insufficiency
- Children with schizophrenia, epilepsy, deafness, metabolic diseases, or other mental conditions
- Use of psychotropic medications or nutritional supplements for symptom management within the previous 3 months
- Hearing impairments or abnormalities
- Presence of intracranial or peristimulation metallic foreign bodies, cardiac pacemakers, or other implanted electronic devices
- Previous receipt of ASD-specific interventions or comprehensive rehabilitation training

### Sample Size

Sample size calculation was based on one-way ANOVA comparing the primary outcome measure (Autism Behavior Checklist [ABC] scores) among the 3 groups. The calculation was performed using the PASS software (version 15; NCSS, LLC) with the following parameters: significance level of α=.05 (2 sided), statistical power of 1 – β = 0.90, and number of groups of *k*=3. On the basis of previous similar studies [33,34], the within-group SD is set at σ=6.5, yielding a medium to large effect size (Cohen *f*). The calculation indicated that a minimum sample size of 15 participants per group was required. Accounting for an anticipated dropout rate of approximately 20%, a total of 20 participants will be recruited for each group. The following standard ANOVA sample size formula was applied: n = ψ^2^ × (σ^2^) / (*k* × *d*^2^).

### Randomization and Allocation Concealment

A researcher (WL) who is not involved in participant recruitment or treatment uses SPSS (version 25.0; IBM Corp) to randomly allocate all eligible participants to 3 groups. The sequence number and group assignment are placed in consecutively numbered, opaque, sealed envelopes. When a participant is enrolled and found to meet the inclusion criteria, the next available envelope is opened to reveal the group allocation, which is then implemented. To ensure balance in terms of important prognostic factors such as age and gender across the intervention groups after randomization, a stratified randomization approach has been adopted. All other researchers involved in this study are blinded to the treatment allocations.

### Intervention Protocol

All participants receive conventional comprehensive rehabilitation treatment, including speech and cognitive training, social skill training, occupational therapy, sensory integration training, and music therapy [35,36], for 30 minutes per session once daily, 5 times per week, for 12 weeks in total.

#### rTMS-Only Group

The rTMS intervention is administered using a CCY-1 TMS device (Yiruide Medical Equipment New Technology Co., Ltd) with a circular coil. Before the first treatment session, the resting motor threshold (RMT) is determined by identifying the cortical representation of the contralateral abductor pollicis brevis muscle within the primary motor cortex (M1). The optimal stimulation site is defined as the location that consistently produces the maximum amplitude (>50 μV) and shortest latency motor-evoked potential in the relaxed abductor pollicis brevis muscle. RMT is defined as the minimum stimulation intensity required to induce motor-evoked potentials of ≥50 μV in at least 5 of 10 consecutive trials [37,38].

During treatment sessions, participants are seated and wear a positioning cap. The bilateral DLPFC is targeted sequentially: the left DLPFC during weeks 1 to 6 and the right DLPFC during weeks 7 to 12 [2]. This sequencing is based on the hypothesis of hemispheric excitation-inhibition imbalance in ASD [39]. The frequency is set to 1 Hz, and the stimulation intensity is set to 80% the RMT with 400 pulses each time at a 20-second interval every 10 pulses 5 times a week for a total of 12 weeks as a course of treatment. Each session lasts approximately 20 minutes. Following rTMS, participants listen to unmodulated natural environmental sounds or music through headphones for an additional 20 minutes to match the total treatment duration of the combined intervention.

#### rTMS Combined With AIT Group

In addition to the standard rehabilitation treatment, participants in this group receive a combination of rTMS and AIT. For the first 6 weeks, the left DLPFC is targeted, followed by right DLPFC stimulation in the subsequent 6 weeks. The 2 treatments are administered simultaneously, with each session consisting of 20 minutes of rTMS followed by 20 minutes of AIT for a total session duration of 40 minutes. The rTMS protocol matches the parameters used in the rTMS-only group.

The AIT intervention is delivered using the Elite RT510 AIT instrument (Shenzhen Elite Medical Equipment Co, Ltd), a digital integrated therapy device. Participants wear headphones during the AIT sessions. The operator selects the appropriate audio filtration settings based on the participant’s age (distinguishing between those over and under 4 years of age) and then plays a series of filtered and modulated music CDs according to the established AIT protocol [40]. The treatment process involves precise control of sound intensity (“sound level”) and attenuation values following a fixed playback sequence. All equipment parameters are reset after each session.

#### Sham rTMS Group

Participants in this group receive conventional comprehensive rehabilitation treatment as well as sham rTMS stimulation using a “sham coil” with the same appearance as that of the active TMS coil. The sham stimulation parameters, including sequence, sound, and frequency, match those of the rTMS-only group. Additionally, during the 20 minutes following the sham rTMS, participants listen to unmodulated and unfiltered natural environmental sounds or music, similar to the rTMS-only group.

### Outcome Measures

#### Primary Outcome Measures

##### ABC Measure

This 57-item scale [41] completed by parents is suitable for screening and assisting in the diagnosis of ASD in individuals aged 8 months to 28 years. It evaluates behavioral symptoms across 5 factors: sensory abilities, communication abilities, motor skills, language, and self-care. Each item is scored on a 4-point scale. A total score of ≥53 indicates a screening cutoff for ASD, whereas a total score of ≥67 indicates a positive diagnosis of ASD.

##### Childhood Autism Rating Scale

This 15-item scale [42] is used to assess symptom severity in children with ASD aged ≥2 years. Qualified personnel will conduct the assessments, with each item scored from 1 to 4. A total score of 30 to 36 with fewer than 5 items with a score below 3 indicates mild to moderate ASD; a total score of >36 with at least 5 items with a score above 3 indicates severe ASD.

### Secondary Outcome Measures

#### Strengths and Difficulties Questionnaire

Developed by Dr Robert Goodman [43], this 25-item questionnaire is used to assess emotional and behavioral problems in individuals aged 2 to 17 years, including those with ASD. The Strengths and Difficulties Questionnaire (SDQ) evaluates 4 areas of difficulty: emotional symptoms, conduct problems, hyperactivity, and peer relationship problems, as well as prosocial behavior. Parents rate their children’s emotional and behavioral performance over the previous 6 months on a 3-point Likert scale (0=“not true,” 1=“somewhat true,” and 2=“certainly true”). Higher scores on the difficulty scales indicate more severe problems, whereas higher scores on the prosocial scale indicate better prosocial behavior. The Cronbach α coefficient is 0.78, demonstrating good reliability and validity.

#### RBS-R Measure

This 43-item scale [44] comprises 6 core components: stereotyped behavior, self-injurious behavior, compulsive behavior, ritualistic behavior, fixated behavior, and restricted behavior. Participants’ parents complete the RBS-R, with higher total scores indicating more severe stereotyped behaviors. The Cronbach α coefficient is 0.85. The Chinese version of this scale has shown good reliability and validity, making it an effective tool for evaluating treatment effects on repetitive behaviors in children with ASD.

### Data Management

Clinical data are collected using a case report form (CRF) provided exclusively by the research team. The CRF serves as the original record and will not be altered. One member of the research team (QH) is responsible for recording and maintaining the CRFs. Upon completion of the trial, 2 researchers (JW and JY) will independently enter the CRF data into the computer for statistical analysis.

### Ethical Considerations

This study has been reviewed and approved by the ethics committee of Longgang Maternal and Child Health Hospital of Shenzhen, China (KYXM-2023-032), in accordance with the Declaration of Helsinki. Parents or legal guardians of child patients who meet the inclusion criteria are informed of the study’s purpose, specific procedures, and potential risks and possible benefits of participation. Those who choose to participate then provide written informed consent. Participants’ personal data will be kept confidential throughout the study and accessible only to the research team. There will be no compensation provided to participants, and no identifiable images of individual participants will be included in the manuscript or supplementary materials.

### Statistical Analysis

Statistical analyses will be conducted using SPSS. All participants, including the first 8 randomized under the original scheme, will be analyzed according to the intention-to-treat principle. At baseline, continuous variables among the 3 groups will be compared using the Kruskal-Wallis test, whereas categorical variables will be compared using the chi-square test to assess the balance of demographic and clinical characteristics (including age, gender, and baseline scores on all outcome measures) across the randomized groups.

Data normality will be assessed, and appropriate statistical tests will be selected accordingly. For normally distributed measurement data, between-group comparisons will be conducted using one-way ANOVA, and within-group pretest-posttest treatment comparisons will be made using paired-sample 2-tailed *t* tests presented as means and SDs. For nonnormally distributed data, nonparametric tests will be used, with results reported as medians and IQRs. *P*<.05 will be considered statistically significant.

In addition to the primary end-point comparisons at study completion, an exploratory interim analysis of outcomes at 6 weeks will be performed to preliminarily assess the effects of left DLPFC stimulation.

### Protocol Amendment

This study randomized the first 8 participants in a 1:1:1 ratio. Subsequently, to more effectively test the core hypothesis (ie, the efficacy of the combined intervention), greater statistical power will be concentrated on the intervention group (rTMS+AIT), and the randomization ratio was ultimately adjusted to 2:1:1.

## Results

### Overview

This study commenced data collection in April 2024. It is anticipated that the rTMS combined with AIT intervention will exhibit superior efficacy compared to rTMS only.

Currently, 8 participants (5 male and 3 female) have been recruited and randomized. Due to the limited sample size, this study has not yet conducted statistical comparisons of baseline characteristics among the 3 groups. The pooled baseline characteristics of the enrolled participants are as follows: mean age of 4.12 years, mean total ABC score of 53.38, and mean total Childhood Autism Rating Scale (CARS) score of 35.25. Recruitment and follow-up of all participants are ongoing.

### Study Timeline and Status

The detailed time points and status of this study are shown in Table 2.

**Table 2.** Study timeline and status notes.

Period	Research activities	Status
January 2024-March 2024	Final protocol development and ethics approval	Completed
April 2024	Recruitment and randomization commenced	From the first participant onward, we consistently used a gender- and age-stratified randomization method.
May 2024-September 2025	Data collection and intervention; protocol refinement	Ongoing. All participants have received interventions per the established protocol. Interim assessments (6 weeks) and baseline evaluations as predefined assessment points have been conducted since study initiation. The first 8 participants were randomized at the initial 1:1:1 ratio. To enhance statistical power for effect size and variability in the combined intervention group (rTMS^a^+AIT^b^), the ratio was ultimately adjusted to 2:1:1.
October 2025-March 2026	Data collection and intervention; recruitment completion	Ongoing; strictly following protocol
March 2026-June 2026	End point assessment; data collation and analysis	Future work

a rTMS: repetitive transcranial magnetic stimulation.

b AIT: auditory integration training.

## Discussion

We hypothesize that, compared to the sham stimulation control group, participants with ASD receiving the active rTMS combined with AIT intervention will exhibit significant improvements in core symptoms such as social difficulties and stereotyped behaviors as measured using standardized scales such as the ABC, CARS, RBS-R, and SDQ. This study aims to provide the latest evidence-based support for the efficacy of this combined intervention approach.

Although preliminary studies have reported positive effects of rTMS combined with AIT in ASD, most have lacked blinding and placebo controls. By implementing a double-blind, sham-controlled design, this trial will address that key methodological limitation. If the results align with our hypotheses, they will provide the first clear demonstration of the specific therapeutic benefits of this combined intervention, thereby advancing the evidence level in this field from the exploratory stage to preliminary validation.

The core strength of this study lies in its rigorous methodological design. As a prospective randomized controlled trial, it uses a double-blind, sham stimulus–controlled approach, which minimizes the potential for expectation and observer biases during the evaluation process, thereby strengthening the causal inferences that can be drawn from the observed efficacy differences. Additionally, the study uses recognized, standardized assessment tools, including the ABC, CARS, SDQ, and RBS-R, to provide a comprehensive and objective evaluation of the core symptom domains in ASD. This assessment combination ensures good reliability and clinical relevance of the study results. Finally, the rTMS and AIT interventions are noninvasive, well tolerated, and easy to implement in clinical settings. These features support participant compliance and trial feasibility and, if the interventions prove effective, will facilitate wider clinical translation as a safe, scalable treatment option for children with ASD.

This study also has some important limitations. First, while rigorous behavioral assessments are used, neurophysiological indicators (eg, EEG and functional near-infrared spectroscopy) are not included. Therefore, this study cannot directly reveal the underlying neural mechanisms of the combined intervention. Future studies should verify these mechanisms in conjunction with neuroimaging techniques. Second, the primary objective is to preliminarily evaluate whether adding AIT to rTMS could yield superior clinical efficacy. As a result, a separate control group receiving AIT alone was not established. Subsequent studies will need to expand the sample size to explore this question. Additionally, the follow-up period after the intervention may be insufficient to assess the long-term durability of the effects, so our conclusions will be limited to discussing the short-term efficacy.

On the basis of the expected results and limitations of this study, future research should focus on the following directions. First, at the mechanistic level, follow-up studies should integrate neuroimaging techniques such as EEG, functional near-infrared spectroscopy, or functional magnetic resonance imaging to directly observe the effects of the combined intervention on brain network connectivity and neural activity. Second, at the level of efficacy optimization, trials with larger sample sizes, longer follow-up periods (eg, 6 months or 1 year), and inclusion of an AIT-only group will be needed to verify the durability of the intervention’s benefits and clarify the individual vs additive benefits of rTMS and AIT.

## Supplementary material

10.2196/80243 Checklist 1 SPIRIT 2025 checklist.

## Abbreviations

ABC: Autism Behavior Checklist
AIT: auditory integration training
ASD: autism spectrum disorder
CARS: Childhood Autism Rating Scale
CRF: case report form
DLPFC: dorsolateral prefrontal cortex
EEG: electroencephalography
RBS-R: Repetitive Behavior Scale–Revised
RMT: resting motor threshold
rTMS: repetitive transcranial magnetic stimulation
SDQ: Strengths and Difficulties Questionnaire
SPIRIT: Standard Protocol Items: Recommendations for Interventional Trials
TMS: transcranial magnetic stimulation
